# Relationship between anatomical characteristics of pulmonary veins and atrial fibrillation recurrence after radiofrequency catheter ablation: a systematic review and meta-analysis

**DOI:** 10.3389/fcvm.2023.1235433

**Published:** 2023-09-19

**Authors:** Dan Qi, Jianjun Zhang

**Affiliations:** Department of Cardiology, Beijing Chaoyang Hospital, Capital Medical University, Beijing, China

**Keywords:** pulmonary veins, atrial fibrillation, radiofrequency catheter ablation, cross-sectional orifices, meta-analysis

## Abstract

**Background:**

The aim of the current study was to investigate the potential relationship between anatomical characteristics of pulmonary veins (PVs) and atrial fibrillation recurrence (AFR) following radiofrequency catheter ablation (RFCA), specifically focusing on PV diameter and cross-sectional orifices index (CSOA). The analysis was based on a comprehensive review of currently available literature, providing valuable insights for the prevention and treatment of AFR.

**Methods:**

Data was collected from five databases, including PubMed, MEDLINE, EMBASE, and Cochrane, spanning the period from 2004 to October 2022. The search strategy utilized Medical Subject Headings (MeSH) terms related to PV diameter, PV size, PV anatomy, and AFR. Indicators of PV diameter and CSOA from the included studies were collected and analyzed, with Weight mean difference (WMD) and 95% confidence intervals (CIs) representing continuous variables.

**Results:**

The meta-analysis included six studies. The results revealed that patients with AFR had a significant larger mean PV diameter compared to those without AFR (MD 0.33; 95% CI: 0.01, 0.66; *P *= 0.04; *I*^2 ^= 33.80%). In a meta-analysis of two studies involving a total of 715 participants, we compared the diameters of the left superior pulmonary vein (LSPV), left inferior pulmonary vein (LIPV), right superior pulmonary vein (RSPV), right inferior pulmonary vein (RIPV) between patients with AFR and patients without AFR. The results showed that there were no statistically significant differences between the two groups in any of the four data items (all *P *> 0.05). Additionally, the pooled estimate revealed that LSPV-CSOA, LIPV-COSA, RSPV-COSA, and RIPV-CSOA were greater in the AFR group compared to the non-AFR group, but the differences were not statistically significant (all *P *> 0.05).

**Conclusion:**

We found evidence supporting the notion that the PV diameter of patients who experienced AFR after RFCA was significantly larger than that of patients without AFR. The findings suggested that the PV diameter could serve as a potential predictor of the risk of AFR following RFCA.

## Introduction

Atrial fibrillation (AF), is the most common type of arrhythmia, and it significantly affects patients’ quality of life. Radiofrequency catheter ablation (RFCA) has emerged as a standard treatment for AF, with pulmonary vein isolation (PVI) being a crucial component of AF-targeting procedure. Despite its widespread use, the cure rate following the initial ablation treatment remains relatively low, with approximately 70% of patients experiencing recurrence in the short term and around half requiring multiple surgeries during long-term follow-up (1). Therefore, there has been considerable effort to predict the benefits of RFCA for individual patients. Previous research has explored various indicators, such as left atrial (LA) diameter, left atrial volume index, and left atrial emptying fraction, to understand their association with atrial fibrillation recurrence (AFR) following RFCA (2–4). Additionally, investigations have focused on the influence of indicators related to the size and anatomical morphology of pulmonary veins (PVs) on AF recurrence after RFCA, including PV diameter and cross-sectional orifices index (CSOA), which can be measured through non-invasive methods like computed tomography (CT). However, the conclusions from retrospective studies regarding whether PV diameter can reliably predict AF recurrence have been inconsistent (5–8).

Herein, our aim was to investigate the relationship between the anatomical characteristics of PV and AFR following RFCA, specifically examining PV diameter and CSOA. We conducted a systematic review and meta-analysis to pool the results from available literature, aiming to provide a theoretical basis for the prevention and treatment of AFR.

## Methods

### Search strategies and data sources

We conducted a comprehensive search of relevant literature from 2004 to October 2022. The databases used for the search included PubMed, MEDLINE, EMBASE, and the Cochrane database. The Preferred Reporting Items for Systematic Reviews and Meta-Analyses (PRISMA) criteria were applied to ensure the systematic and transparent approach in conducting this meta-analysis (9). The search strategy utilized Medical Subject Headings (MeSH) terms, which included pulmonary vein: pulmonary vein diameter, pulmonary vein size, left-superior pulmonary vein, left-inferior pulmonary vein, right-superior pulmonary vein, right-inferior pulmonary vein, cross-sectional orifices, and atrial fibrillation recurrence. (Supplementary Table S1) Additionally, we carefully examined the reference lists of the included publications to identify any other relevant studies.

### Inclusion and exclusion criteria

For this meta-analysis, we considered all studies that investigated the relationship between preoperative anatomical characteristics of PV and AFR after RFCA in patients with AF. Only the most recent data from the same study was included if it was published in multiple journals, ensuring the accuracy and consistency of the results.

The criteria for inclusion were as follows: (1) The study design should be either prospective or retrospective. (2) Study participants should be 18 years of age or older. (3) RFCA should be based on PVI. (4) PV size should be assessed using CT. (5) The studies should have a follow-up period longer than 6 months after RFCA. (6) The studies should use univariate or multivariate regression analysis to determine the probability of AFR following RFCA based on per unit increase in PV size. (7) At each follow-up appointment, ambulatory Holter monitoring should be performed for 24 h to detect symptomatic or asymptomatic recurrences. (8) The articles should be written in English or Chinese.

The criteria for exclusion were as follows: (1) Studies that lacked a control group were excluded. (2) Studies that compared mean PV in patients with AFR after direct current cardioversion or cryoballoon ablation were excluded. (3) Animal tests and reviews were not considered. (4) Studies that only investigated the connection between PV and AFR in protocol or abstract form were not included.

### Data extraction and assessment of study quality

Two reviewers evenly extracted all relevant data components from each included study. The extracted data included the first author's name, year of publication, sample size, mean value in each group, standard deviation (SD) in each group, details of the ablation process, type of AF (paroxysmal or persistent), detection method, duration of follow-up, and recurrence detection. In case of any disagreements between the reviewers, a joint reevaluation of the original article was conducted.

### Quality assessment

The quality of each study was independently assessed by two reviewers using the Cochrane risk of bias tool, which evaluates various aspects such as random sequence generation, allocation concealment, blinding of participants and personnel, blinding of outcome assessment, incomplete outcome data, selective reporting, and other bias. To examine possible publication bias, funnel plots were generated, and formal statistical testing using the Egger and Begger tests was performed.

### Statistical analysis

Weight mean difference (WMD) and 95% confidence intervals (CIs) were utilized to represent continuous variables. Heterogeneity among the studies was assessed using the *Q*-statistical test and the *I*^2^ test, with a *P* value < 0.10 or *I*^2^ > 50% indicating considerable heterogeneity. The *I*^2^ statistic quantified the percentage of variability in the trials that could be attributable to heterogeneity rather than chance. Studies with *I*^2^ statistic of 25%, 50%, 75%, and 100% were categorized as having no, low, moderate, and high heterogeneity, respectively (10). When significant heterogeneity was identified, a random-effects model was used to pool the data. Otherwise, a fixed-effects model was applied. Sensitivity analysis was conducted by omitting individual studies one by one to determine their impact on the overall results. Publication bias was evaluated using funnel plots or the Egger and Begger tests (11). A *P* value less than 0.05 was considered statistically significant, unless otherwise specified. The data were analyzed using Stata version 16.0.

## Results

### Study selection

**Figure 1:**
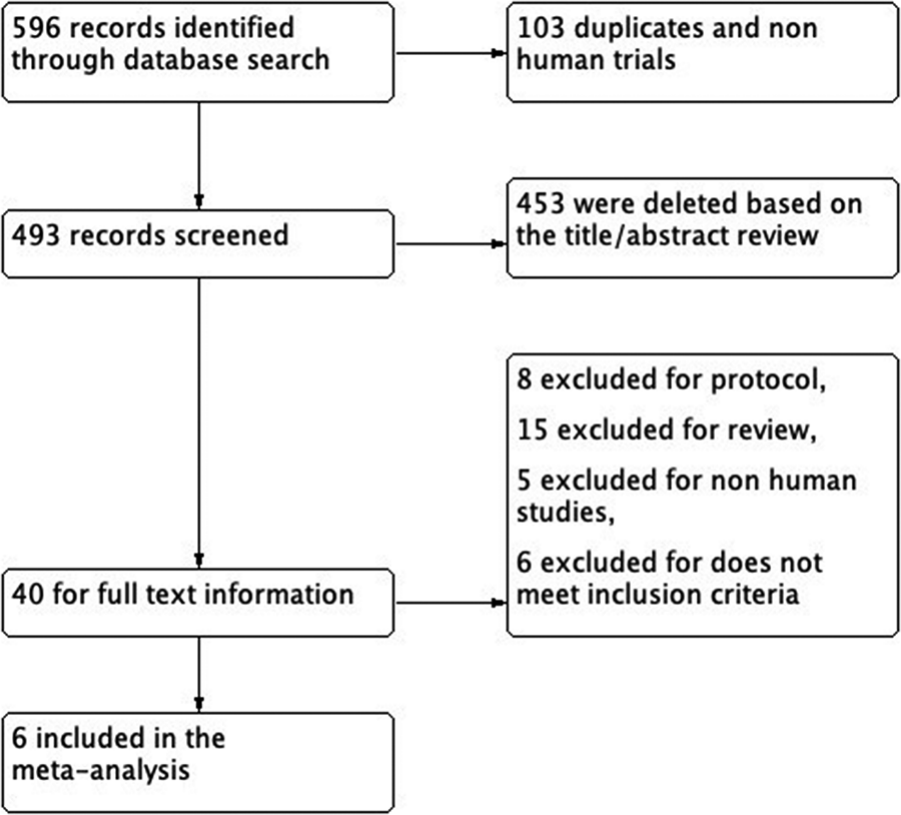
Flowchart of the literature selection.

Initially, our search yielded 596 relevant publications, and after further review, 493 entries were considered for the next step. Subsequently, 453 entries were eliminated based on the examination of their titles and abstracts. The full-text content of 40 publications was then thoroughly reviewed. Following the application of the inclusion and exclusion criteria, 6 studies were ultimately selected for the meta-analysis (Figure 1).

### Study characteristics

Table 1 illustrated the baseline characteristics of the 6 included studies (7, 12–16). These studies, published between 2011 and 2022, had a total sample size of 1,226 participants. The average follow-up period in these investigations was 15.78 months.

**Table 1:** Baseline characteristics of the six included studies.

Writer	Year	Sample size	Ablation procedure	AF		Detection method	Follow-up (months)	Recurrence detection	AFR number
				Paroxysmal	Persistent				
Dennis, W	2011	100	PVI	72	28	CT	11.6 ± 2.8	24h-Holter	35
Alex J. A. McLellan	2014	102	PVI	102	0	CT	12 ± 4	7day-Holter	27
Wei wei	2014	267	PVI	210	57	CT	10	24h-Holter	44
Shimamoto, K	2018	118	PVI	73		CT	14	24h-Holter	23
				45					19
Lee, W. C	2019	191	PVI	191	0	CT	12	24h-Holter	31
Szegedi, N.	2021	448	PVI	345	103	CT	24	24h-Holter	120

AFR, recurrence of atrial fibrillation; PVI, pulmonary vein isolation; CT, computed tomography.

For all included patients, electroanatomical imaging of the left atrium and pulmonary veins was completed under the guidance of a three-dimensional mapping system and ablation was performed based on the electroanatomical imaging obtained. The ablation energy was provided by a saline-irrigated ablation catheter, and the ablation temperature was set to be lower than 43°C. Circumferential pulmonary vein ablation was employed under the guidance of the Carto system. The default power was set at 35–40 W.

All the studies utilized circumferential PVI alone. The correlation values of PV anatomical features were measured using CT in all the studies. Moreover, Holter monitoring was employed in all the investigations to diagnose the recurrence of asymptomatic AF.

### Quality of studies

The majority of the studies were of high quality. The most notable bias addressed was related to the selection of special populations and other interventions. The Egger's test indicated that there was no publication bias.

### Difference in preoperative PV diameter between AFR and non-AFR patients after RFCA

**Figure 2:**
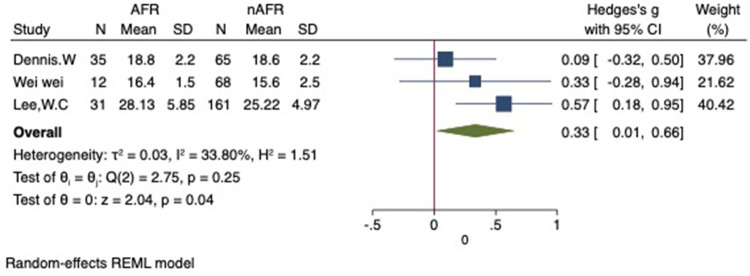
Forest plot of difference in PV diameter between AFR patients and non-AFR patients. PV, pulmonary vein; AFR, recurrence of atrial fibrillation.

**Figure 3:**
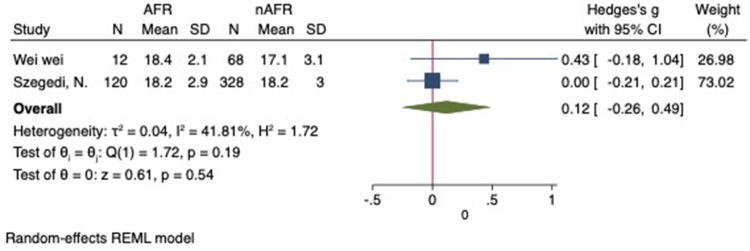
Forest plot of difference in LSPV diameter between AFR patients and non-AFR patients. LSPV, left-superior pulmonary vein; AFR, recurrence of atrial fibrillation.

**Figure 4:**
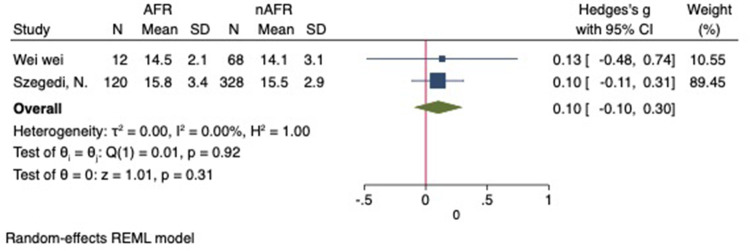
Forest plot of difference in LIPV diameter between AFR patients and non-AFR patients. LIPV, left-inferior pulmonary vein; AFR, recurrence of atrial fibrillation.

**Figure 5:**
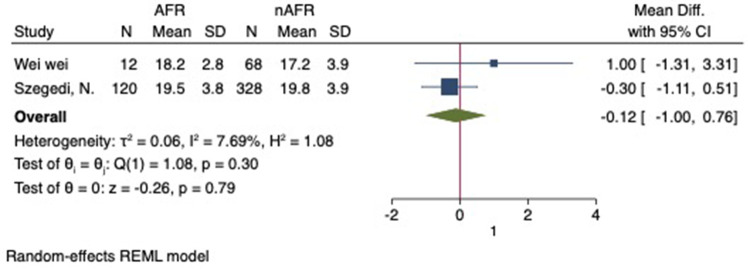
Forest plot of difference in RSPV diameter between AFR patients and non-AFR patients. RSPV, right-superior pulmonary vein; AFR, recurrence of atrial fibrillation.

**Figure 6:**
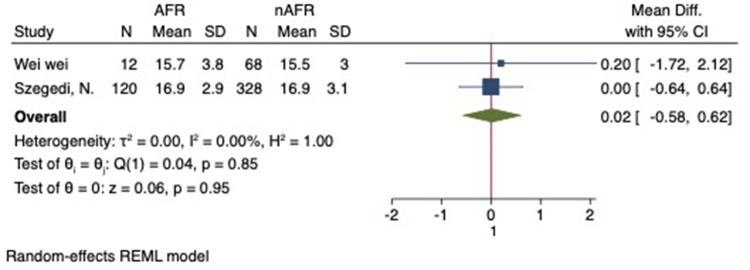
Forest plot of difference in RIPV diameter between AFR patients and non-AFR patients. RIPV, right-inferior pulmonary vein; AFR, recurrence of atrial fibrillation.

**Figure 7:**
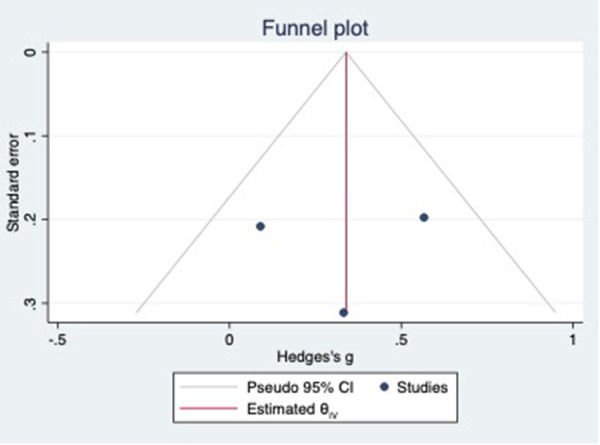
Funnel plot showing the publication bias in PV diameter.

For this meta-analysis, three studies with a total of 558 participants were included to compare the difference in PV diameter between AFR patients and those without AFR (7, 12, 13). The pooled results demonstrated a significantly higher mean PV diameter in the AFR group compared to the non-AFR group. (MD: 0.33; 95% CI: 0.01, 0.66; *P *= 0.04; *I*^2 ^= 33.80%) (Figure 2). Additionally, 2 studies, involving a total of 715 participants, were included in the meta-analysis, which showed no statistically significant differences in diameters of left-superior pulmonary vein (LSPV), left-inferior pulmonary vein (LIPV), right-superior pulmonary vein (RSPV) and right-inferior pulmonary vein (RIPV) between AFR patients and non-AFR patients (LSPV-MD: 0.12; 95% CI: −0.26, 0.49; *P *= 0.54; *I*^2 ^= 41.81%) (LIPV-MD: 0.10; 95% CI: −0.10, 0.30; *P *= 0.31; *I*^2 ^= 0%) (RSPV-MD: −0.03; 95% CI: −0.26, 0.19; *P *= 0.78; *I*^2 ^= 6.85%) (RIPV-MD: 0.01; 95% CI: −0.19, 0.20; *P *= 0.95; *I*^2 ^= 0%) (Figures 3–6) (12, 14). The funnel plot indicated symmetry, suggesting no publication bias (Figure 7). Furthermore, two studies provided the cut-off values of PV diameter for predicting AFR. Lee et al. reported a cut-off of 25.5 mm (13), while Wei et al. reported the cut-offs of 16.1 mm for LSPV, 14.0 mm for LIPV, 16.2 mm for RSPV, and 17.8 mm for the RIPV (12).

### Difference in preoperative PV-CSOA between AFR and non-AFR patients after RFCA

**Figure 8:**
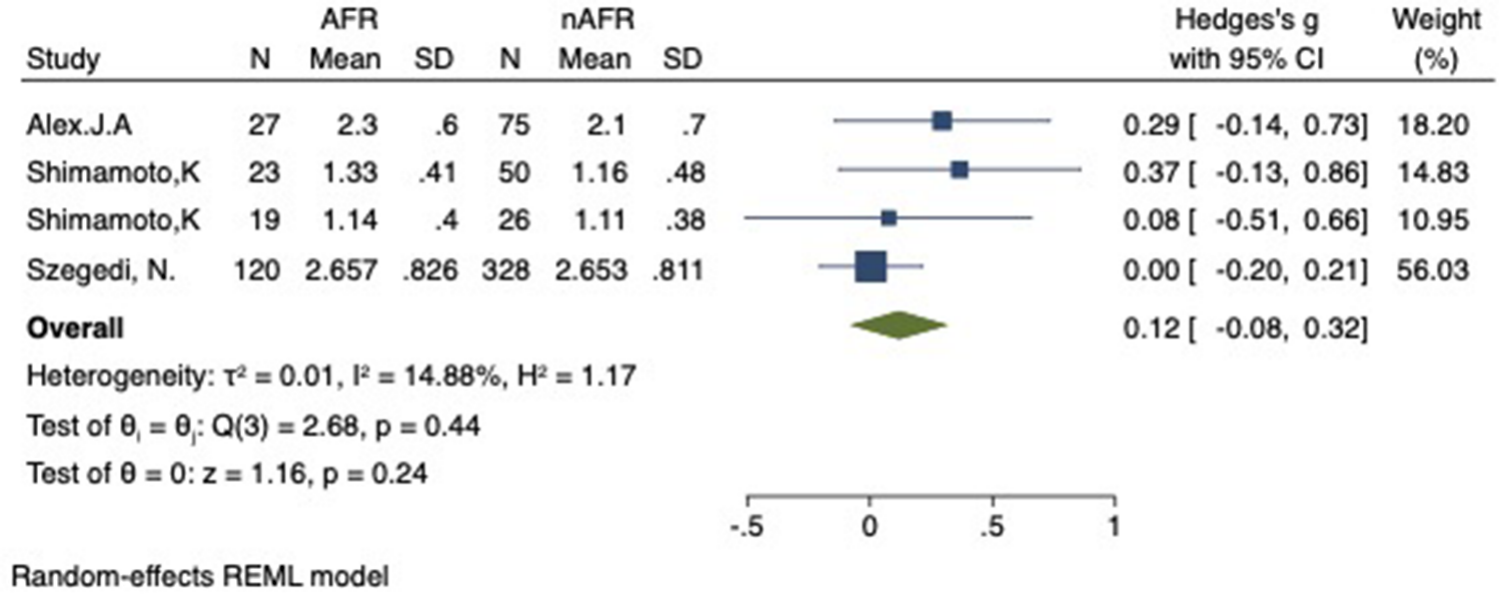
Forest plot of difference in LSPV-CSOA between AFR and non-AFR patients. LSPV, left-superior pulmonary vein; CSOA, cross-sectional orifices index; AFR, recurrence of atrial fibrillation.

**Figure 9:**
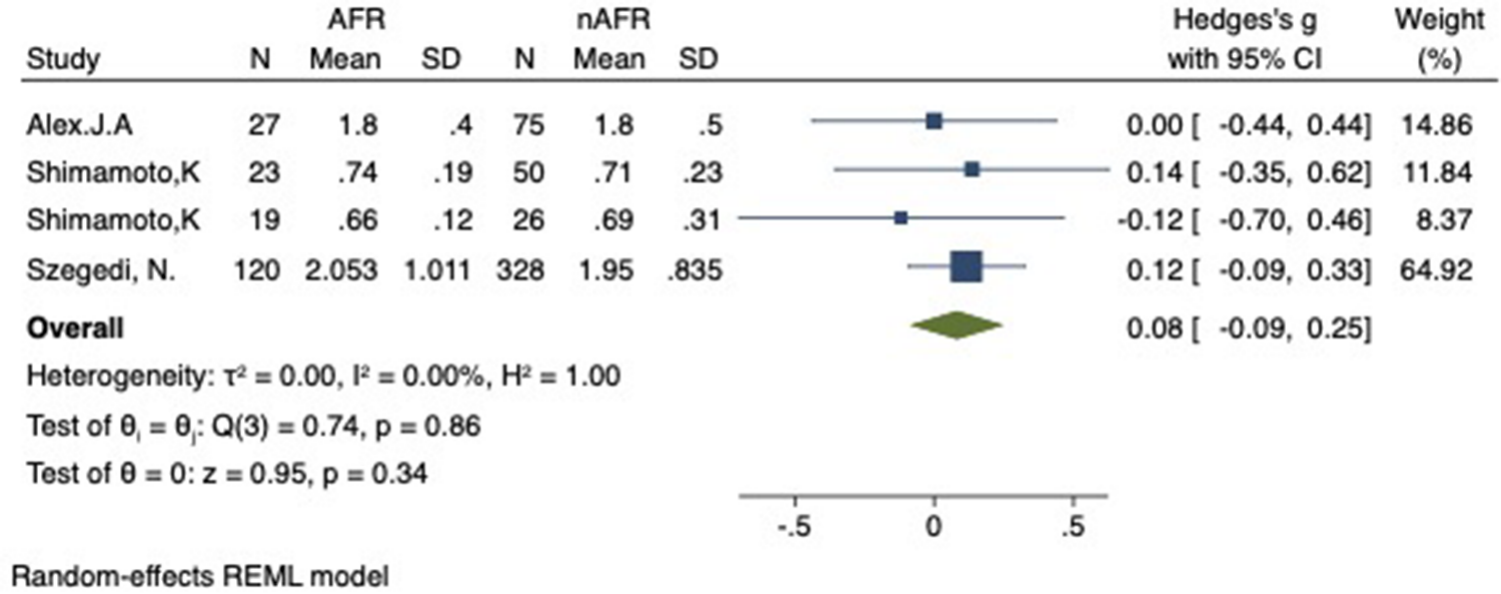
Forest plot of difference in LIPV-CSOA between AFR and non-AFR patients. LIPV, left-inferior pulmonary vein; CSOA, cross-sectional orifices index; AFR, recurrence of atrial fibrillation.

**Figure 10:**
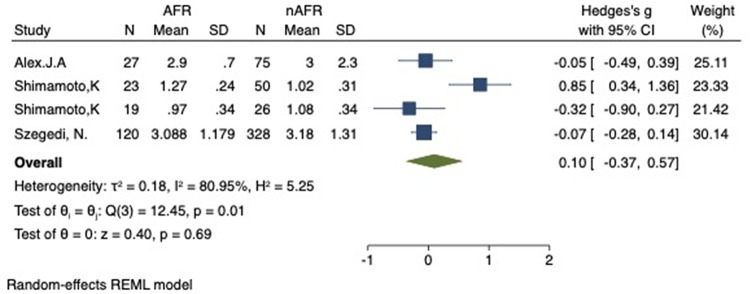
Forest plot of difference in RSPV-CSOA between AFR and non-AFR patients. RSPV, right-superior pulmonary vein; CSOA, cross-sectional orifices index; AFR, recurrence of atrial fibrillation.

**Figure 11:**
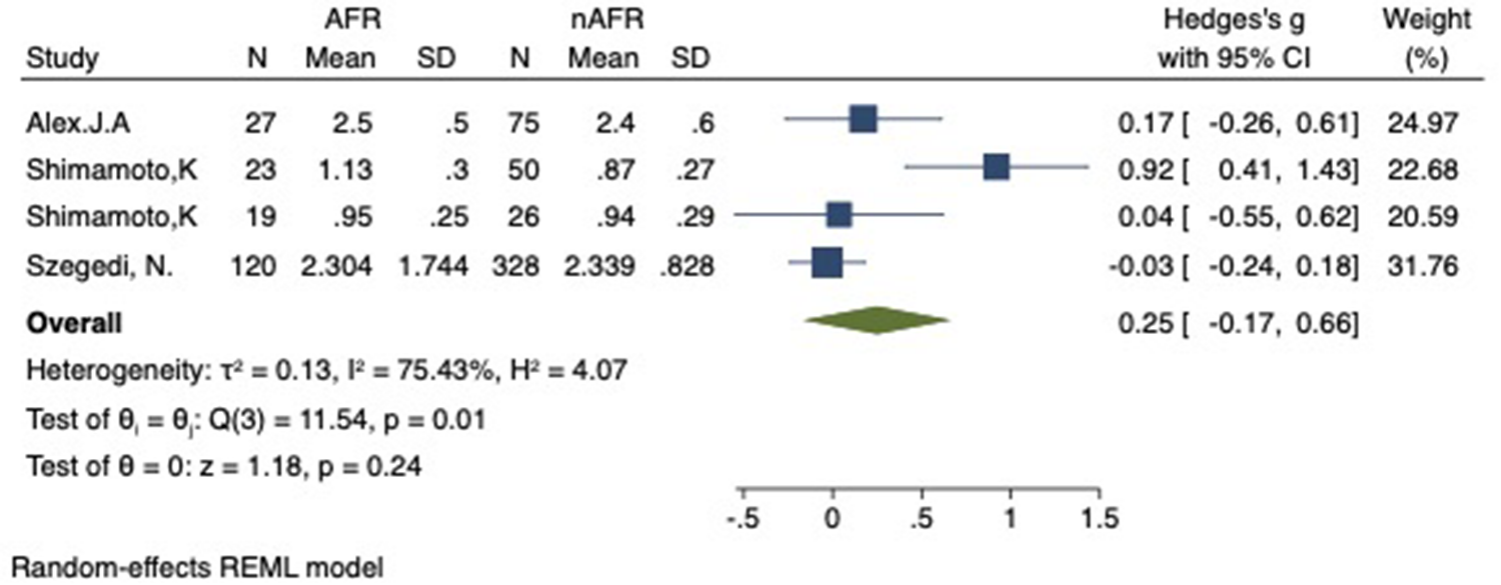
Forest plot of difference in RIPV-CSOA between AFR and non-AFR patients. RIPV, right-inferior pulmonary vein; CSOA, cross-sectional orifices index; AFR, recurrence of atrial fibrillation.

**Figure 12:**
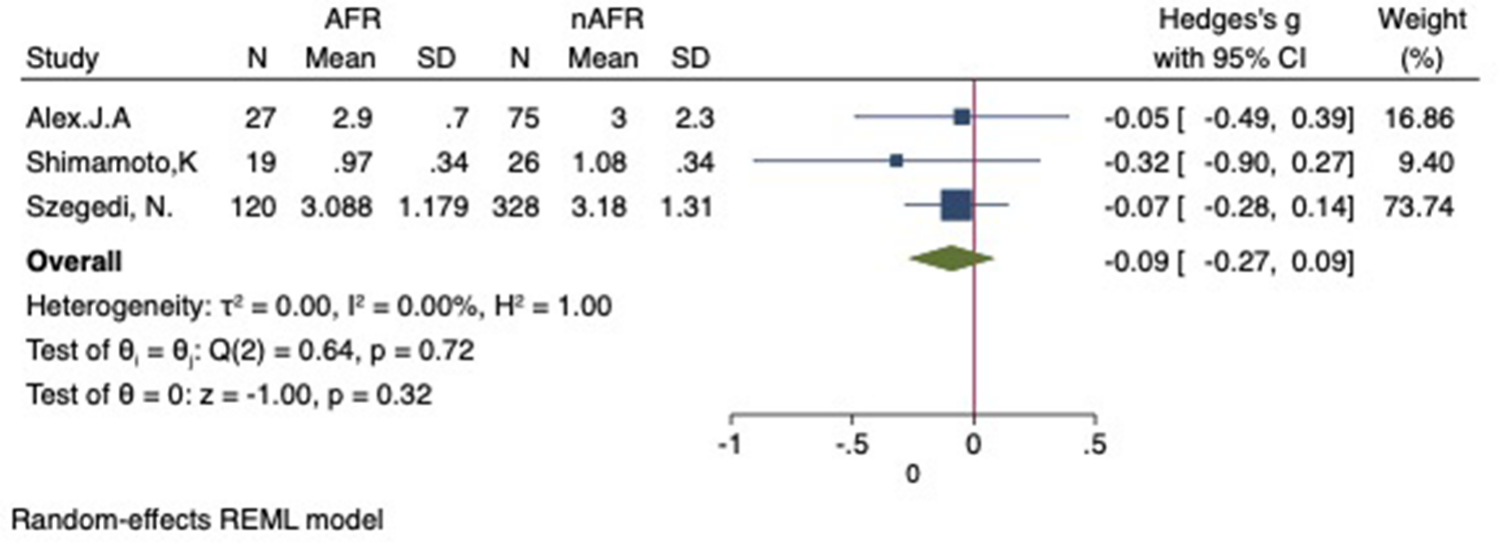
Forest plot for the sensitivity analysis of the difference in RSPV-CSOA between AFR and non-AFR patients. The result was reliable. RSPV, right-suferior pulmonary vein; CSOA, cross-sectional orifices index; AFR, recurrence of atrial fibrillation.

**Figure 13:**
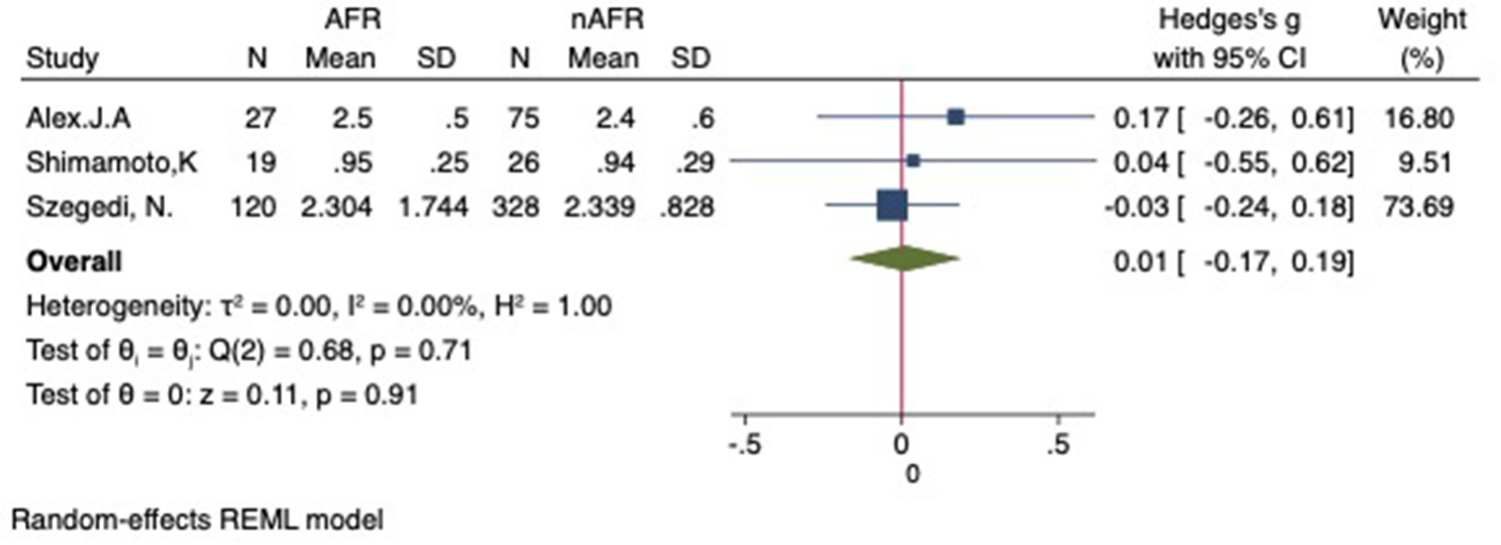
Forest plot for the sensitivity analysis of the difference in RIPV-CSOA between AFR and non-AFR patients. The result was reliable. RIPV, right-inferior pulmonary vein; CSOA, cross-sectional orifices index; AFR, recurrence of atrial fibrillation.

**Figure 14:**
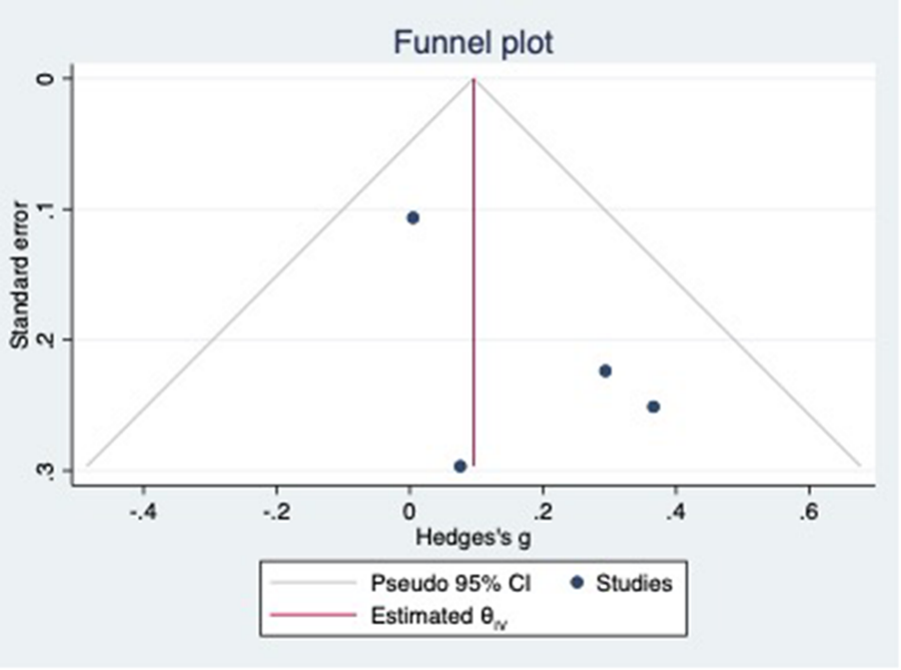
Funnel plot showing the publication bias in PV-COSA.

For this meta-analysis, three studies with a total of 668 participants were included to compare the difference in PV-CSOA between AFR patients and non-AFR patients (14–16). PV-CSOA was expressed as an index divided by the body surface area for left superior (LSPV-CSOA), left inferior (LIPV-CSOA), right superior (RSPV-CSOA), and right inferior (RIPV-CSOA). The pooled estimate showed that these four indexed were greater in the AFR group than in the non-AFR group, but the differences were not statistically significant (LSPV-CSOA: 0.12; 95% CI: −0.08, 0.32; *P *= 0.24; *I*^2 ^= 14.88%) (LIPV-CSOA: 0.08; 95% CI: −0.09, 0.25; *P *= 0.34; *I*^2 ^= 0%) (RSPV-CSOA: 0.10; 95% CI: −0.37, 0.57; *P *= 0.69; *I*^2 ^= 80.95%) (RIPV-CSOA: 0.25; 95% CI: −0.17, 0.66; *P *= 0.24; *I*^2 ^= 75.43%) (Figures 8–11). A separate sensitivity analysis was conducted, where the study by Shimamoto et al., which excluded patients based on RSPV and RIPV, was excluded from the analysis. The results of this sensitivity analysis did not alter the overall inference of the meta-analysis (RSPV-MD: −0.09; CI: −0.27, 0.09; *P = *0.32; RIPV-MD: 0.01; CI: −0.17, 0.19; *P = *0.91). however, it was observed that the heterogeneity among the studies was reduced (*I*^2 ^= 0%) (Figures 12, 13). The funnel plot exhibited a symmetrical distribution of data points, indicating no evidence of publication bias (Figure 14).

## Discussion

In recent years, percutaneous RFCA, combined with medications, has witnessed rapid advancements in the treatment of AF and has emerged as one of the most crucial therapeutic options for managing AF. Among the various approaches, RFCA based on PVI remains the most common treatment for AF (17). However, despite its popularity, the success rate of RFCA for AF varies significantly worldwide, and there is a certain risk of recurrence. This variability in outcomes could be linked to differences in operation methods, patient characteristics, and evaluation criteria across different patient populations. Several studies in recent years have explored various factors that may be associated with AFR after RFCA, such as age, obesity, large left atrial, organic heart disease, type of AF, and duration of AF, to better evaluate the outcomes of RFCA for AF treatment (18–23). PV and the left atrium share common origins from the original common PV and have similar anatomical and histological features. As a result, the characteristics and manifestations of PV may play a significant role in indicating the occurrence and recurrence of AF (24).

The association between PV architecture and AFR has been a topic of debate. In this study, we conducted a unique meta-analysis based on the most recent data to examine the relevance of PV deformation in predicting AFR in well-defined populations undergoing RFCA. To the best of our knowledge, this study was the first meta-analysis on this specific topic. The results of our meta-analysis revealed that, AFR patients, after RFCA, had a significantly larger mean PV diameter compared to non-AFR patients, and these measures may independently be associated with significantly elevated risks of AF recurrence. Notably, our findings implied that the mean difference in PV diameter between individuals with and without AFR was relatively small, about 0.33.

Previous studies have reported on enlarged PV in patients with AF (25). However, the relationship between PV anatomy and the outcome of RFCA for AF remains largely unknown. Some studies showed that enlarged PV diameter was an independent predictor of postoperative AFR (12). However, other studies with relatively large sample sizes yielded inconsistent results regarding the use of PV diameter as a predictor of AFR after RFA. Our findings aligned with the majority of previous investigations, emphasizing the importance of incorporating these indicators into routine clinical practice. The individual PV-CSOA, divided by the body surface area, showed differences between AFR and non-AFR patients, but the differences were not statistically significant. Nevertheless, a large PV size may still be associated with a considerably higher risk of AF recurrence. In comparison to other typical classical metrics such as LA diameter and LA volume index, our findings asserted that PV size provided a novel strategy in predicting AFR.

Numerous studies have been conducted to investigate the potential involvement and mechanisms of PV in the incidence, development, and postoperative AFR. However, isolating electrical activity of PVs from the left atrial is technically challenging due to their large PV size. The development of an insufficient lesion can elevate the probability of LA-PV reconnection, which constitutes the most prevalent cause of AFR (26). Patients with larger PVs may require longer and larger ablation lesions that include a portion of the PV ostium. This makes it more challenging to generate a permanent, transmural, and contiguous lesion, thereby increasing the risk of LA-PV electrical reconnection and AFR (27). Furthermore, a large PV size may exhibit histological and electrophysiological abnormalities, creating an aberrant substrate that is susceptible to AFR (28). The mechanism of postoperative AF recurrence is complex, and additional studies are warranted to further explore it. Cardiac fibrosis was found to be related to AFR in some studies, and a reduction in LA strain was linked to pathological changes in the LA wall tissue and the degree of fibrosis, as determined by advanced gadolinium-enhanced magnetic resonance imaging (29). Besides fibrosis, other factors were shown to contribute to the AFR after RFCA surgery, including atrial fat infiltration, inflammatory infiltration, necrosis, and amyloid deposition (30, 31). Earlier studies also observed delayed damage recovery in late AFR following PVI (32).

Non-invasive methods, such as magnetic resonance imaging (MRI) and multidetector computed tomography (MDCT), along with invasive procedures, such as transesophageal echocardiography (TEE) and intracardiac echocardiography (ICE), have been applied to measure the size of the PVs (33–37). Among these, MDCT and MRI are the most commonly utilized imaging approaches to guide RFCA for AF. ICE is widely employed in the transseptal technique and has demonstrated its ability to reduce complications during AF surgeries, while providing real-time measurements of PV-CSOA (38, 39). Nakashima, T et al. indicated that ICE could be a viable alternative imaging method for assessing PV-CSOA during RFCA, and the index of LSPV-CSOA was found to be a useful independent predictor of AF recurrence following RFCA (40). The LSPV-CSOA cutoff value of 154 mm^2^/m^2^ had a 50% positive predictive value and an 89% negative predictive value for AFR (40). The findings of our meta-analysis supported the results of the abovementioned study. Different studies applied various measurements for strain estimates, as revealed in our systematic review. Subgroup analyses should be performed to evaluate the impact of these different measures on AFR; however, the data available were restricted. The factor of PV variant was not included in the analysis in this study, and a previous study showed that the atypical right middle pulmonary vein was not associated with AFR (7).

Although this study represented the first meta-analysis investigating the impact of anatomical characteristics of PV on AFR after RFCA, it is essential to acknowledge the limitations in our research. Firstly, the included studies primarily consisted of single-center observational studies with small sample sizes, lacking prospective RCTs, which could potentially introduce bias. Secondly, we did not differentiate between patients with paroxysmal AF and persistent AF, thereby reducing the generalizability of the conclusions. It is worth noting that patients with persistent AF were more susceptible to AFR, and this association may be independent of PV anatomy. Thirdly, the limited number of collected independent variable indicators may have affected the comprehensiveness of the regression analysis results. As a result, we lacked sufficient data to ascertain whether PV diameter could be regarded as an independent factor causing AFR, irrespective of variables such as LA diameter and PV reconnections.

## Conclusion

We demonstrated that the PV diameter in patients with AFR after RFCA was significantly larger compared to patients without AFR. This findings suggested that PV diameter could serve as a potential predictor of the risk of AFR following RFCA.

## Data Availability

The original contributions presented in the study are included in the article/**Supplementary Material**, further inquiries can be directed to the corresponding author.
