# Supplementary material for: Relationship between anatomical characteristics of pulmonary veins and atrial fibrillation recurrence after radiofrequency catheter ablation: a systematic review and meta-analysis
Source: Front Cardiovasc Med. 2023 Sep 19;10:1235433. doi: 10.3389/fcvm.2023.1235433 (PMC10546190; doi:10.3389/fcvm.2023.1235433)
Supplement: Supplementary file 1 [file Table1.docx]

**Supplementary Table 1 Searching queries in each database**

| Pubmed | #1 “pulmonary vein” [MeSH Terms]  #2 “Pulmonary vein diameter” [All Fields]  #3 “pulmonary vein size” [All Fields]  #4 “left-superior pulmonary vein” [All Fields]  #5 “left-inferior pulmonary vein” [All Fields]  #6“right-superior pulmonary vein” [All Fields]  #7“right-inferior pulmonary vein” [All Fields]  #8 “cross-sectional orifices” [MeSH Terms]  #9 #1 OR #2 OR #3 OR #4 OR #5 OR #6 OR #7 OR #8  #10 “atrial fibrillation” [MeSH Terms]  #11 “atrial fibrillation recurrence”[All Fields]  #12 #10 OR #11  #13 #9 AND #12 |
| --- | --- |
| MEDLINE | #1 “pulmonary vein” [MeSH Terms]  #2 “Pulmonary vein diameter” [All Fields]  #3 “pulmonary vein size” [All Fields]  #4 “left-superior pulmonary vein” [All Fields]  #5 “left-inferior pulmonary vein” [All Fields]  #6“right-superior pulmonary vein” [All Fields]  #7“right-inferior pulmonary vein” [All Fields]  #8 “cross-sectional orifices” [MeSH Terms]  #9 #1 OR #2 OR #3 OR #4 OR #5 OR #6 OR #7 OR #8  #10 “atrial fibrillation” [MeSH Terms]  #11 “atrial fibrillation recurrence”[All Fields]  #12 #10 OR #11  #13 #9 AND #12 |
| Embase | #1 ‘pulmonary vein’/exp  #2 ‘Pulmonary vein diameter’ti, ab,kw OR ‘pulmonary vein size’ti, ab, kw OR ‘left-superior pulmonary vein’ti, ab, kw OR ‘left-inferior pulmonary vein’ti, ab, kw OR ‘right-superior pulmonary vein’ti,ab,kw OR ‘right-inferior pulmonary vein’ti,ab,kw OR ‘cross-sectional orifices’ti,ab,kw  #3 #1 OR #2  #4 ‘atrial fibrillation’/exp  #5 ‘atrial fibrillation recurrence’ti,ab.kw  #6 #4 OR #5  #7 #3 AND #6 |
| Cochrane | #1 MeSH descriptor: [Pulmonary vein] this term only  #2 Pulmonary vein diameter OR pulmonary vein size OR left-superior pulmonary vein OR left-inferior pulmonary vein OR right-superior pulmonary vein OR right-inferior pulmonary vein OR cross-sectional orifices: ti, ab, kw  #3 #1 OR #2  #4 atrial fibrillation OR atrial fibrillation recurrence: ti, ab, kw  #5 #3 AND #4 |
